# Involvement of ayu NOD2 in NF-κB and MAPK signaling pathways: Insights into functional conservation of NOD2 in antibacterial innate immunity

**DOI:** 10.24272/j.issn.2095-8137.2018.066

**Published:** 2018-05-25

**Authors:** Yi Ren, Shui-Fang Liu, Li Nie, Shi-Yu Cai, Jiong Chen

**Affiliations:** 1 Laboratory of Biochemistry and Molecular Biology, School of Marine Sciences, Ningbo University, Ningbo Zhejiang 315211, China; 2 Key Laboratory of Applied Marine Biotechnology of Ministry of Education, Ningbo University, Ningbo Zhejiang 315211, China

**Keywords:** Ayu NOD2, NF-κB signaling, MAPK signaling, Inflammatory cytokines, *Vibrio anguillarum* infection

## Abstract

Nucleotide oligomerization domain 2 (NOD2) is a major cytoplasmic sensor for pathogens and is critical for the clearance of cytosolic bacteria in mammals. However, studies regarding NOD2, especially the initiated signaling pathways, are scarce in teleost species. In this study, we identified a NOD2 molecule (PaNOD2) from ayu (*Plecoglossus altivelis*). Bioinformatics analysis showed the structure of NOD2 to be highly conserved during vertebrate evolution. Dual-luciferase reporter assays examined the activation of NF-κB signaling and Western blotting analysis detected the phosphorylation of three MAP kinases (p-38, Erk1/2, and JNK1/2). Functional study revealed that, like its mammalian counterparts, PaNOD2 was the receptor of the bacterial cell wall component muramyl dipeptide (MDP), and the leucine-rich repeat motif was responsible for the recognition and binding of PaNOD2 with the ligand. Overexpression of PaNOD2 activated the NF-κB signaling pathway, leading to the upregulation of inflammatory cytokines, including TNF-α and IL-1β in HEK293T cells and ayu head kidney-derived monocytes/macrophages (MO/MΦ). Particularly, we found that PaNOD2 activated the MAPK signaling pathways, as indicated by the increased phosphorylation of p-38, Erk1/2, and JNK1/2, which have not been characterized in any teleost species previously. Our findings proved that the NOD2 molecule and initiated pathways are conserved between mammals and ayu. Therefore, ayu could be used as an animal model to investigate NOD2-based diseases and therapeutic applications.

## INTRODUCTION

Microorganisms that invade a host are initially recognized and cleared by the innate immune system and related immune responses (Medzhitov, 2007a). Innate immune responses are the first line of host defense and are primed upon recognizing pathogen-associated molecular patterns (PAMPs) by germline-encoded pattern recognition receptors (PRRs). Several classes of PRRs, including Toll-like receptors, retinoic acid-inducible gene I (RIG-I)-like receptors, nucleotide oligomerization domain (NOD)-like receptors (NLRs), and cytosolic viral DNA sensors, recognize distinct microbial components and directly activate immune cells (Brubaker et al., 2015; Keating et al., 2011; Medzhitov, 2007b; Sabbah et al., 2009; Yoneyama et al., 2004). Exposure of these receptors to their corresponding PAMPs activates intracellular signaling cascades that induce the expression of a variety of inflammatory-related genes and cytokines.

Nucleotide oligomerization domain 2 (NOD2), a member of the NLR family, is a major cytoplasmic sensor for a peptidoglycan fragment existing in both gram-positive and gram-negative bacteria, named muramyl dipeptide (MDP) (Kufer et al., 2006). Structurally, NOD2 contains two N-terminal tandem caspase activation and recruitment domains (CARDs), a central nucleotide-binding domain (NBD), and multiple C-terminal leucine-rich repeats (LRRs). In resting cells, NOD2 is held in an autoinhibited monomeric state by intramolecular inhibition of the NBD domain through the LRR motifs. After recognizing MDP by LRRs, NOD2 oligomerizes through its NBD domain and recruits the downstream adaptor receptor-interacting serine/threonine kinase 2 (RIPK2) by homotypic CARD-CARD interactions to activate NF-κB and mitogen-activated protein kinase (MAPK) signaling pathways, thus inducing a series of immune responses (Girardin et al., 2003; Tanabe et al., 2004; Windheim et al., 2007). These pathways are essential for bacterial clearance, the disruption of which increases host susceptibility to various kinds of pathogens.

Despite the substantial information about NOD2 in mammals, knowledge of this molecule in lower vertebrates, especially in teleosts, remains limited. Compared with other teleost species, studies on NOD2 in zebrafish (*Danio rerio*) are the most comprehensive. For example, the structural model of the NACHT domain and ATP binding orientations, antibacterial and protective functions, induced NF-κB signaling pathways, and mutual regulation between NOD2 and RIG-I signaling in zebrafish have been well studied (Maharana et al., 2015; Nie et al., 2017; Oehlers et al., 2011; Zou et al., 2016). Other than zebrafish, preliminary studies on NOD2 in other teleosts, including grass carp (*Ctenopharyngodon idella*), Nile tilapia (*Oreochromis niloticus*), Indian major carp (*Catla catla*), rohu (*Labeo rohita*), orange-spotted grouper (*Epinephelus coioides*), miiuy croaker (*Miichthys miiuy*), and rainbow trout (*Oncorhynchus mykiss*) have been carried out (Basu et al., 2016; Chang et al., 2011; Chen et al., 2010; Gao et al., 2018; Hou et al., 2012; Li et al., 2015; Maharana et al., 2013). However, most studies have focused on bioinformatics analysis of this protein, tissue expression levels and inflammatory gene expression levels under different stress. Hence, comprehensive study regarding signaling pathways, especially whether teleost NOD2 can also activate MAPK signaling pathways like in mammals, is required.

Ayu (*Plecoglossus altivelis*) is an economically important and widely cultured fish in East Asia. However, the development of the ayu aquaculture is challenged by bacterial and viral fish diseases that have caused production and animal welfare problems (Zhang et al., 2015). Given the importance of NOD2 in antibacterial innate immune responses, studying the ligands, functions, and induced signaling pathways of ayu NOD2 is important. In the present study, we identified a NOD2 gene from ayu (PaNOD2), which, like its mammalian counterparts, contains all functional domains. The mRNA expression patterns of PaNOD2 were examined in various tissues under normal conditions and after *Vibrio anguillarum* challenge. The subcellular localization and ligands of PaNOD2 were analyzed. Importantly, we demonstrated the conserved involvement of PaNOD2 in the NF-κB and MAPK signaling pathways. This study will hopefully enrich current understanding of teleost NLRs in antibacterial immunity and provide valuable insights into the evolutionary history of cytosolic PRRs in innate immunity from teleosts to mammals.

## MATERIALS AND METHODS

### Experimental fish

All experimental ayu (45.0±2.4 g, *n*=250) used in the present study were purchased from a fishery in Ninghai County, Ningbo City, China. Healthy fish were temporarily maintained in a recirculating water system (21.0±1.0 ${}^{\circ }$C) with regular feeding, as described previously (Zhang et al., 2018). The fish were acclimatized to laboratory conditions for two weeks before the experiments. All experiments were performed according to the Experimental Animal Management Law of China and approved by the Animal Ethics Committee of Ningbo University.

### Molecular characterization of PaNOD2

The cDNA sequence of PaNOD2 was retrieved from the transcriptome data of the ayu head kidney-derived monocytes/macrophages (MO/MΦ). Specific primers were designed to amplify the open reading frame (ORF) of PaNOD2 using reverse transcription PCR (NOD2 F1/R1, Table 1), followed by cloning and sequencing. Multiple sequence alignment was performed using ClustalW with homologous sequences retrieved by BLAST (http://blast.ncbi.nlm.nih.gov/Blast.cgi). Functional motifs in proteins were analyzed using the SMART (http://smart.embl-heidelberg.de/) and Pfam databases (http://smart.embl-heidelberg.de/; http://pfam.xfam.org/) (Letunic et al., 2012). The phylogenies of the protein sequences were estimated with MEGA 5.0 software using parsimony and the neighbor-joining method (Tamura et al., 2011). Sequences used in this study are listed in Table 2.

**Table 1:** Oligonucleotide primers used in this work

Primer	Gene	GenBank accession No.	Nucleotide sequence (5${}^{\prime }$–3${}^{\prime }$)	Amplicon size (bp)	Usage of primer pairs
NOD2 F1	*PaNOD2*	MG674830	ATGAGTGCCCAGCAGTTGGTGCTAAG	2 964	Cloning of PaNOD2
NOD2 R1			TCAGAACGTTAGTCGGGA		
NOD2 F2	*PaNOD2*	MG674830	GAATTCATGAGTGCCCAGCAGTTGGTGCTAA	2 964	Construct EGFP fusion plasmid
NOD2 R2			GGATCCCGGAACGTTAGTCGGGACTCT		
NOD2 F3	*PaNOD2*	MG674830	ATGCGCGGCCGCTATGAGTGCCCAGCAGTTGGTGCTAA	2 964	Construct eukaryotic expression plasmid
NOD2 R3	*PaNOD2*	MG674830	ATGCGCGATCGCGAACGTTAGTCGGGACTCT		
NOD2 R4	*PaNOD2*	MG674830	ATGCGCGATCGCGATTGCCAGAGGAACGATGGCG	2 205	Construct ΔLRR mutant
NOD2 qRT F	*PaNOD2*	MG674830	GGATGACATTTACACCGAAGG	244	Quantification of NOD2 gene expression
NOD2 qRT R			TCTGCGACAACTGAATGGA		
TNF-α qRT F	*PaTNF-*α	JP740414	ACATGGGAGCTGTGTTCCTC	115	Quantification of TNF-α gene expression
TNF-α qRT R			GCAAACACACCGAAAAAGGT		
IL-1β qRT F	*PaIL-1*β	HF543937	TACCGGTTGGTACATCAGCA	104	Quantification of IL-1β gene expression
IL-1β qRT R			TGACGGTAAAGTTGGTGCAA		
18S rRNA qRT F	*Pa18S rRNA*	FN646593	GAATGTCTGCCCTATCAACT	103	Quantification of 18S rRNA gene expression
18S rRNA qRT R			GATGTGGTAGCCGTTTCT		

**Table 2:** NOD1 and NOD2 sequences used in this study

GenBank accession No.	Species		Protein
	Latin name	English name	
MG674830	*Plecoglossus altivelis*	Ayu	NOD2
XP_022597963.1	*Seriola dumerili*	Amberjack	NOD2
XP_022522840.1	*Astyanax mexicanus*	Mexican tetra	NOD2
ERE77544.1	*Cricetulus griseus*	Chinese hamster	NOD2
XP_020797036.1	*Boleophthalmus pectinirostris*	Mudskipper	NOD2
XP_015236187.1	*Cyprinodon variegatus*	Sheepshead minnows	NOD2
XP_008335431.1	*Cynoglossus semilaevis*	Half-smooth tongue sole	NOD2
XP_012715032.1	*Fundulus heteroclitus*	Killifish	NOD2
ACX71753.1	*Ctenopharyngodon idella*	Grass carp	NOD2
AFV53358.1	*Epinephelus coioides*	Orange-spotted grouper	NOD2
AEG89706.1	*Labeo rohita*	Rohu	NOD2
AKR76246.1	*Miichthys miiuy*	Miiuy croaker	NOD2
XP_003437591.1	*Oreochromis niloticus*	Nile tilapia	NOD2
XP_014031576.1	*Salmo salar*	Atlantic salmon	NOD2
ADV31549.1	*Oncorhynchus mykiss*	Rainbow trout	NOD2a
ADV31550.1	*Oncorhynchus mykiss*	Rainbow trout	NOD2b
XP_017314821.1	*Ictalurus punctatus*	Channel catfish	NOD2
XP_018522174.1	*Larimichthys crocea*	Large yellow croaker	NOD2
XP_020481540.1	*Labrus bergylta*	Ballan wrasse	NOD2
NP_001314973.1	*Danio rerio*	Zebrafish	NOD2
XP_017548715.1	*Pygocentrus nattereri*	Red-bellied piranhas	NOD2
XP_019935411.1	*Paralichthys olivaceus*	Japanese flounder	NOD2
NP_001035913.1	*Takifugu rubripes*	Pufferfish	NOD2
NP_001002889.1	*Bos taurus*	Cattle	NOD2
NP_665856.2	*Mus musculus*	Mouse	NOD2
NP_001280486.1	*Homo sapiens*	Human	NOD2
XP_020792937.1	*Boleophthalmus pectinirostris*	Mudskipper	NOD1
XP_004571362.1	*Maylandia zebra*	Zebra mbuna	NOD1
XP_002665106.3	*Danio rerio*	Zebrafish	NOD1
AII73558.1	*Oncorhynchus mykiss*	Rainbow trout	NOD1
XP_018418247.1	*Nanorana parkeri*	Tibetan frog	NOD1
NP_001002889.1	*Bos taurus*	Cattle	NOD1
NP_001164478.1	*Mus musculus*	Mouse	NOD1
NP_006083.1	*Homo sapiens*	Human	NOD1

### Plasmid constructions

The ORF of PaNOD2 was inserted into EGPF-N1 (Clontech, USA) between the *Eco*R I and *Bam*H I sites to construct EGFP fusion proteins (pEGFP-PaNOD2, NOD2 F2/R2), and was inserted into pcDNA3.1-FLAG (kind gift from Prof. Zong-Ping Xia) between *Not* I and *Sfa*A I to construct eukaryotic expression vectors (pcDNA3.1-PaNOD2, NOD2 F3/R3). The LRR motif-deleted mutant (ΔLRR) constructs [pcDNA3.1-PaNOD2 (ΔLRR), NOD2 F3/R4] were cloned and inserted into pcDNA3.1-FLAG between the *Not* I and *Sfa*A I sites. The NF-κB luciferase construct was purchased from Clontech (Palo Alto, USA) and the pRL-TK vector was obtained from Promega (Madison, USA). All constructed sequences were confirmed by sequencing analysis. The plasmids for transfection were prepared at the endotoxin-free level using an EZNA™ Plasmid Midi Kit (Omega Bio-Tek, USA).

### *In vivo* bacterial challenge and expression analysis of PaNOD2

The *V. anguillarum* challenge was carried out as reported previously (Zhang et al., 2018). Briefly, *V. anguillarum* were grown at 28 ${}^{\circ }$C in nutrient broth and harvested at the logarithmic phase before washing, resuspending, and diluting to the appropriate concentration in sterile phosphate-buffered saline (PBS). The final bacterial concentration was confirmed by plating serial dilutions on solid media. Each ayu was intraperitoneally injected with 1.2×10^4^ colony forming units (CFUs) of live *V. anguillarum* (in 100 μL PBS), with PBS injected alone used as the control group. Ayu were sacrificed at 4, 8, 12, 24, or 48 h post-infection (hpi) and the gill, spleen, head kidney, liver, and intestine were collected for total RNA extraction (Chen et al., 2018). The RNA of healthy fish tissues, including the muscle, skin, heart, gill, spleen, head kidney, liver, and intestine, were also extracted for tissue expression pattern analysis. Real-time quantitative PCR (RT-qPCR) was performed on an ABI StepOne Real-Time PCR System (Applied Biosystems, USA) using SYBR premix Ex Taq II (TaKaRa, Japan) with the following protocol: (1) 40 cycles of amplification at 95 ${}^{\circ }$C for 30 s and 60 ${}^{\circ }$C for 20 s; (2) melting curve analysis at 95 ${}^{\circ }$C for 5 s, 65 ${}^{\circ }$C for 15 s, and 95 ${}^{\circ }$C for 15 s; and (3) cooling at 40 ${}^{\circ }$C for 30 s. Relative gene expression was calculated using the 2^−ΔΔCT^ method with PaNOD2 initially normalized against Pa18S rRNA. The primers used are listed in Table 1 (NOD2 qRT F/R). Each PCR trial was performed in triplicate and repeated at least three times.

### Cell culture and transient transfection

Ayu head kidney-derived MO/MΦ were isolated as described previously (He et al., 2013). The isolated MO/MΦ were seeded into 35-mm dishes (2×10^7^/mL) and cultured in RPMI 1640 medium (Invitrogen, China) supplemented with 5% (v/v) fetal bovine serum (FBS) (Gibco, Life Technologies, USA), 5% (v/v) ayu serum, penicillin (100 U/mL), and streptomycin (100 μg/mL) at 24 ${}^{\circ }$C in 5% CO_2_ after washing off non-adherent cells. The HEK293T cells were maintained in Dulbecco’s modified Eagle’s medium (DMEM, Invitrogen, China) supplemented with 10% (v/v) FBS, penicillin (100 U/mL), and streptomycin (100 μg/mL) at 37 ${}^{\circ }$C in 5% CO_2_. The HEK293T cells (1×10^5^/mL) were seeded into multi-well plates (Corning, USA) to allow growth until 70%–90% confluence on the day of transfection. Both cell types were transiently transfected with DNA using lipofectamine 3000 (Invitrogen, China) according to the manufacturer’s instructions.

### Subcellular localization

The HEK293T cells were cultured and seeded onto cover slips in 6-well plates before transfection. After 24 h of culture, the cells were transfected with pEGFP-PaNOD2 (1 μg/mL). At 36 h post-transfection (hpt), the cells were washed twice with PBS and fixed for 20 min in 4% (v/v) paraformaldehyde, followed by staining with DiI (Beyotime, China) for 10 min. After twice washing with PBS, the nuclei were stained with DAPI (Sigma, USA) for 5 min. Fluorescence images of cells were obtained using a confocal microscope (Zeiss LSM710 NLO, Carl Zeiss, Germany).

### Dual-luciferase report assay

The HEK293T cells and ayu MO/MΦ were transfected with relative plasmids (1 μg/mL) and NF-κB luciferase reporter vectors (200 pg/mL). The pRL-TK Renilla luciferase reporter plasmid was used as an internal control. The empty control plasmid pcDNA3.1-FLAG was added to ensure the same amounts of total DNA. Cells were lysed at 24 hpt and dual-luciferase reporter assay was performed (Nie et al., 2015). Luciferase activity was normalized to pRL-TK activity and expressed as fold stimulation relative to the control.

### Role of PaNOD2 in activating NF-κB signaling pathway

NF-κB activation was examined in both HEK293T cells and ayu MO/MΦ using luciferase assay and RT-qPCR according to above description. For luciferase assay, pcDNA3.1-FLAG, pcDNA3.1-PaNOD2, or pcDNA3.1-PaNOD2 (ΔLRR), together with the NF-κB luciferase reporter gene, were transfected into both cell types, with the pRL-TK Renilla luciferase reporter plasmid used as an internal control. At 24 hpt, cells were harvested and firefly and Renilla luciferase activities were assayed with three replicates according to the manufacturer’s instructions. Luciferase activity was normalized to pRL-TK activity and expressed as fold stimulation relative to the control. For RT-qPCR, pcDNA3.1-FLAG or pcDNA3.1-PaNOD2 (1 μg/mL) were transfected into HEK293T cells or ayu MO/MΦ and the expression of TNF-α and IL-1β were analyzed at 6, 12, and 24 hpt. Each trial was performed in triplicate and repeated at least three times. Results were displayed relative to the corresponding Pa18S rRNA values to calculate relative copy numbers.

### Recognition assay

As HEK293T cells do not express endogenous NOD2, they are the perfect system to investigate the ligands of PaNOD2. The HEK293T cells were transfected with pcDNA3.1-PaNOD2 (1 μg/mL) or MDP (10 ng/mL) or both with PaNOD2 at gradient levels, together with NF-κB luciferase reporter vector. The pRL-TK Renilla luciferase reporter plasmid was used as the internal control. Empty control plasmid pcDNA3.1-FLAG was added to ensure the same amounts of total DNA. At 24 hpt, cells were lysed, and dual-luciferase reporter assay was conducted, as described above.

### Role of PaNOD2 in activating MAPK signaling pathways

Both cell types were transfected with pcDNA3.1-FLAG or pcDNA3.1-PaNOD2 (1 μg/mL). At 12, 24, 36, and 48 hpt, the activation of the MAPK signaling pathways was examined by Western blotting using antibodies against three representative MAP kinases (p-38, ERK1/2, and JNK1/2). The primary antibodies used were rabbit phospho-p38 MAPK (Thr180/Tyr182), rabbit p38 MAPK, rabbit phospho-SAPK/JNK1/2 (Thr183/Tyr185), rabbit SAPK/JNK1/2, rabbit phospho-p44/42 MAPK (ERK1/2) (Thr202/Tyr204), and rabbit p44/42 MAPK (ERK1/2) (Cell Signaling Technology, China). The secondary antibody used was HRP-conjugated goat anti-rabbit IgG (Life Technologies, China). The blot was then incubated with enhanced chemiluminescence (ECL) reagents (Life Technologies, China) according to the manufacturer’s protocols.

### Statistical analyses

Data from three independent experiments were expressed as means±*SEM*. Statistical analysis was conducted by one-way analysis of variance (ANOVA) with SPSS version 13.0 (SPSS Inc, Chicago, USA). *P*<0.05 and *P*<0.01 were considered statistically significant.

## RESULTS

### Molecular characterization of PaNOD2

**Figure 1:**
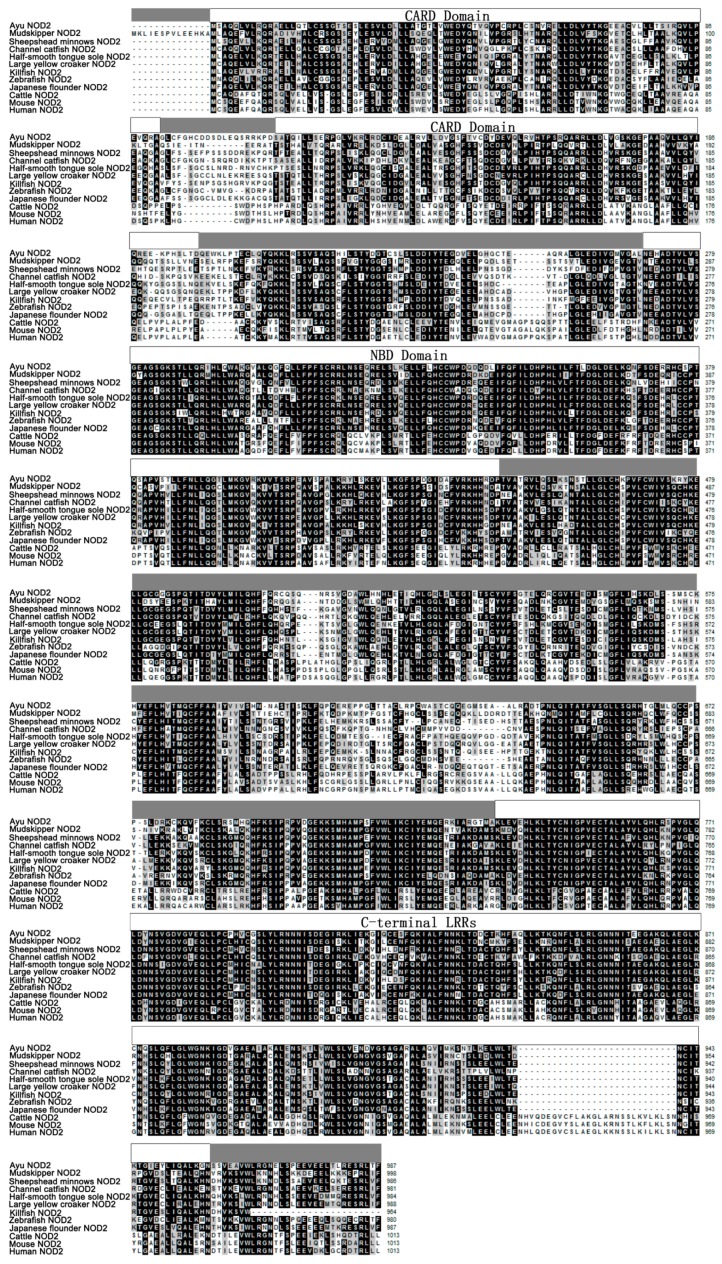
Multiple sequence alignment of PaNOD2 with other homologues

**Figure 2:**
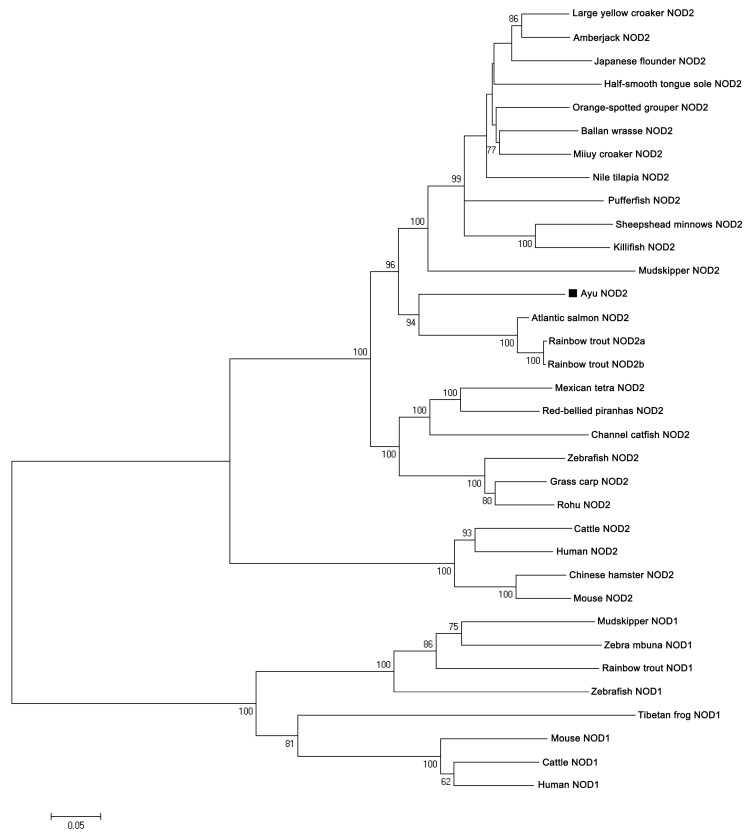
Phylogenetic tree showing relationship of PaNOD2 with other known NOD2 homologues and other NLR family members using MEGA 5.0

The ORF of PaNOD2 was 2 964 bp in length and encoded a protein with 987 amino acids. The molecular weight (MW) of the protein was 1.1036×10^5^ and the putative isoelectric point (*p*I) was 6.20. The PaNOD2 protein also contained two N-terminal tandem CARD domains, central NBD domain, and multiple C-terminal LRRs. Multiple sequence alignment showed that the protein sequence and domains of NOD2 were conserved among vertebrates, and PaNOD2 shared highest amino acid identity and similarity (67% identity and 81% similarity) with the large yellow croaker homolog (Figure 1). Phylogenetic tree analysis showed that all teleost NOD2 proteins were clustered together with their higher vertebrate counterparts and formed a distinct group with the NOD1 proteins, which also belonged to the NLR family (Figure 2).

The threshold for shading is >60%; similar residues are marked with gray shading, identical residues are marked with black shading, and alignment gaps are marked as “–”. Accession numbers of sequences used are listed in Table 2. Two N-terminal tandem CARD domains, central NBD domain, and multiple C-terminal LRRs are indicated above the alignment.

Values at forks indicate the percentage of trees in which this grouping occurred after bootstrapping (1 000 replicates; shown only when >60%). Scale bar shows number of substitutions per base. GenBank accession Nos. of sequences are listed in Table 2. The ayu NOD2 site is marked with ◾.

### Tissue distribution and expression analysis of PaNOD2

**Figure 3:**
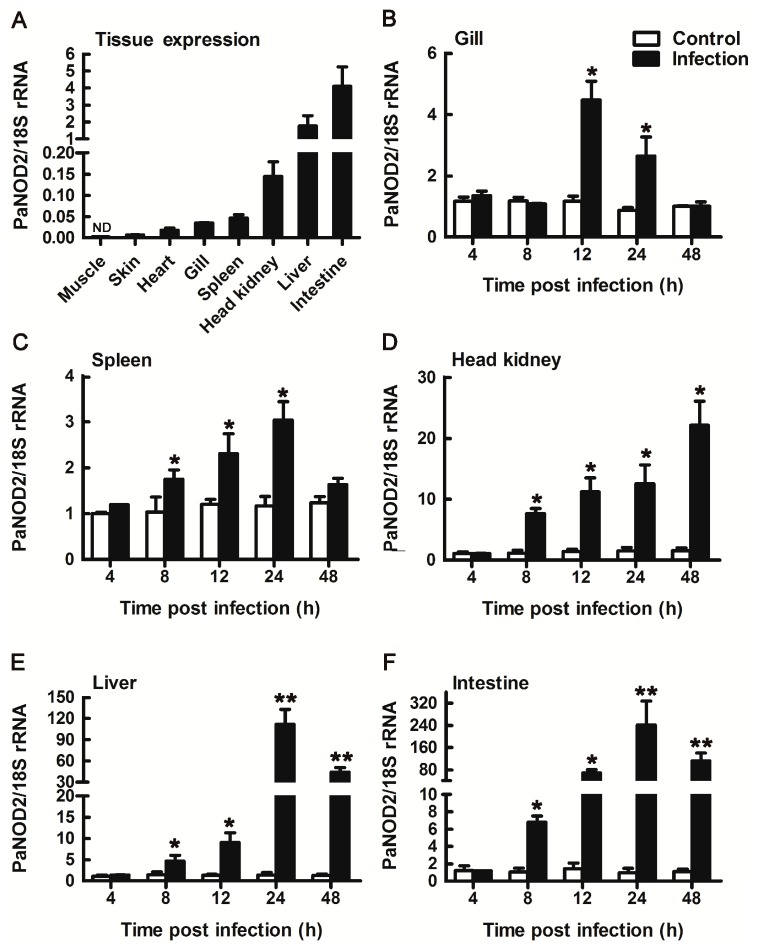
RT-qPCR analysis of PaNOD2 expression patterns in healthy ayu tissues and immune tissues after *V. anguillarum* infection

The mRNA expression pattern of PaNOD2 was detected in the skin, heart, gill, spleen, head kidney, liver, and intestine of ayu. The highest expression of PaNOD2 was observed in the intestine, followed by the liver and head kidney (Figure 3A). Upon *V. anguillarum* infection, PaNOD2 mRNA expression was upregulated in all examined immune-related tissues in a time-dependent manner. The PaNOD2 transcript in the gill was dramatically upregulated at 12 hpi, then gradually decreased and returned to normal status at 48 hpi (Figure 3B). In the spleen, head kidney, liver, and intestine, PaNOD2 expression was upregulated at 8 hpi and gradually increased as the infection continued. The highest expression in the spleen, liver, and intestine was observed at 24 hpi, whereas that in the head kidney was detected at 48 hpi (Figure 3B–F).

A: Relative PaNOD2 expression in various healthy ayu tissues (muscle, skin, heart, gill, spleen, head kidney, liver, intestine) against 18S rRNA, ND is not detected. Relative expression was calculated as the average of three replicates, each consisting of four fish samples. B–F: PaNOD2 transcripts in immune tissues of ayu challenged with *V. anguillarum*. Tissues were collected at 4, 8, 12, 24, and 48 hpi. Relative PaNOD2 transcript levels to 18S rRNA were quantified, and the mRNA level in the 4-h PBS-injected group was normalized to 1. Data are means±*SEM* of results from three replicates *: *P*<0.05, **: *P*<0.01.

### Subcellular localization of PaNOD2

**Figure 4:**
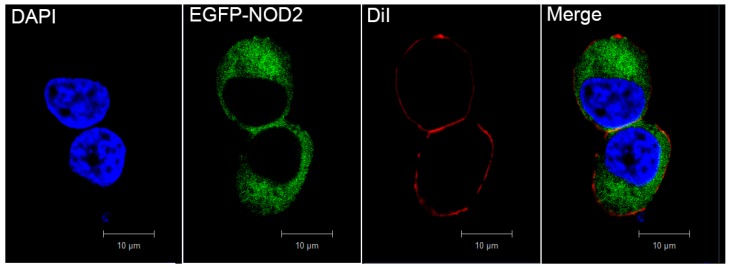
Subcellular localization of PaNOD2

Before functional characterization, subcellular localization of PaNOD2 was initially evaluated by introducing an EGFP-fused construct (EGFP-PaNOD2) into the HEK293T cells. PaNOD2 was clearly distributed in the cytoplasm of the transfected cells, with no colocalized signals with DiI (membrane indicator) or DAPI (nucleus indicator) (Figure 4).

Cytoplasmic localization of PaNOD2 in HEK293T cells observed using confocal microscopy. Nucleus was stained by DAPI and cell membrane was stained by DiI. Scale bars: 10 μm, original magnification ×640.

### Role of PaNOD2 in activating NF-κB signaling pathway

**Figure 5:**
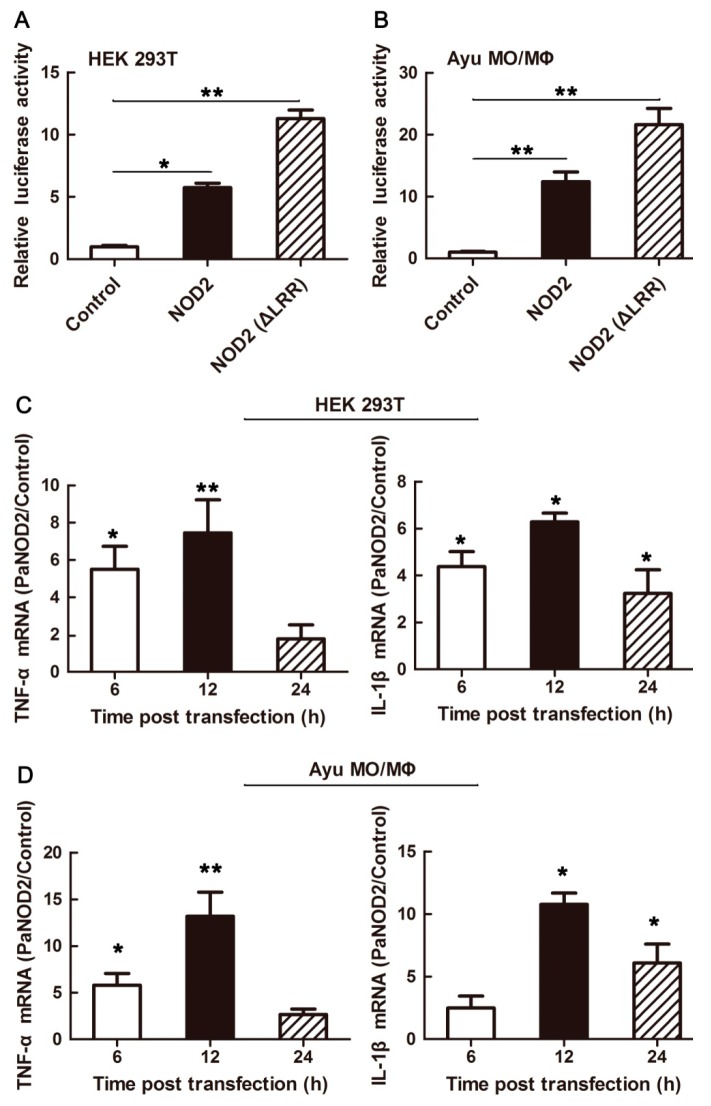
Activation of NF-κB signaling pathway by PaNOD2 and LRR-deleted mutant PaNOD2 (ΔLRR)

As an important pathway initiated by NOD2 signaling, activation of the NF-κB signaling pathway was examined to provide evidence for the conserved role of PaNOD2 in antibacterial immunity. Luciferase reporter activity and inflammatory cytokine (TNF-α and IL-1β) expression were detected to evaluate the activation of the NF-κB signaling pathway. Results showed that overexpression of PaNOD2 in both the HEK293T cells and ayu MO/MΦ for 24 h significantly (*P*<0.05 and *P*<0.01) induced NF-κB activation, as determined by the dual-luciferase report assay (Figure 5A, B). In addition, the expressions of TNF-α and IL-1β were also upregulated in the PaNOD2 overexpression group (Figure 5C, D). The LRR-deleted mutant PaNOD2 (ΔLRR) construct was used to examine whether the LRR domain in PaNOD2 acted to keep this molecule in an autoinhibited status. As expected, overexpression of the ΔLRR mutant in both HEK293T cells and ayu MO/MΦ greatly enhanced the extent of NF-κB activation compared with the wild group (Figure 5A, B). Clearly, PaNOD2 played a conserved role in the NF-κB signaling pathway and the LRR structure acted as an autoinhibition domain, which was regulated by recognition of the receptor to bacterial PAMPs.

HEK293T cells (A) and ayu MO/MΦ (B) were transfected with pcDNA3.1-PaNOD2 or pcDNA3.1-PaNOD2 (ΔLRR), together with the NF-κB luciferase reporter vector, with an empty plasmid transfected group used as the control. Luciferase reporter assays were conducted at 24 hpt. C, D: Examination of the expression of inflammatory cytokines (TNF-α and IL-1β). HEK293T cells (C) and ayu MO/MΦ (D) were transfected with pcDNA3.1-PaNOD2 or empty plasmid, with cells collected at 6, 12, and 24 hpt for RNA extraction and real-time PCR to measure the expression of TNF-α and IL-1β. Values are means±*SEM*; *: *P*<0.05, **: *P*<0.01.

### Recognition of PaNOD2 to bacterial PAMPs

**Figure 6:**
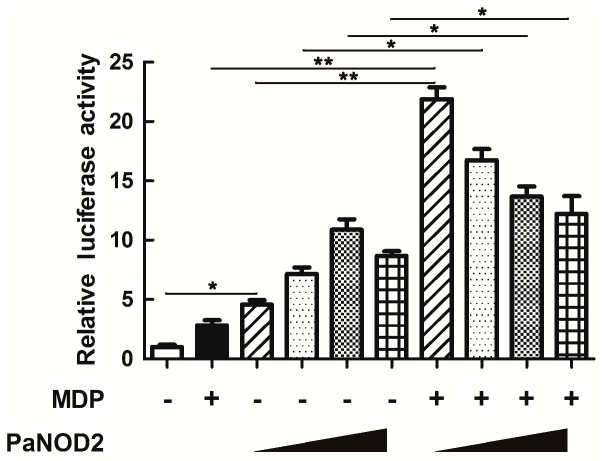
Functional evaluation of PaNOD2 as a receptor of MDP

For recognition analysis, HEK293T cells (with no endogenous expression of NOD2) were used for ectopic expression of PaNOD2; and MDP, a typical PAMP molecule on the cell walls of both gram-negative and gram-positive bacteria, was used for stimulation of PaNOD2-dependent NF-κB activation. Results showed that activation of NF-κB could be induced (*P*<0.05) with increased administration of the PaNOD2-expression vector and significantly higher with the co-administration of MDP (*P*<0.05 and *P*<0.01, Figure 6). Thus, PaNOD2 is an intracellular PRR participating in the recognition of bacterial MDP, whose performance is similar to that of mammalian NOD2s.

Non-endogenous NOD2-expression HEK293T cells were used for this purpose. HEK293T cells were transfected with pcDNA3.1-PaNOD2 or MDP (10 ng/mL) or both with PaNOD2 at gradient levels (up to 1 μg/mL), together with NF-κB luciferase reporter vector. Empty-plasmid transfected group was used as the control. Luciferase reporter assays were conducted at 24 hpt. Values are means±*SEM*; *: *P*<0.05, **: *P*<0.01.

### Conserved role of PaNOD2 in activating MAPK signaling pathways

**Figure 7:**
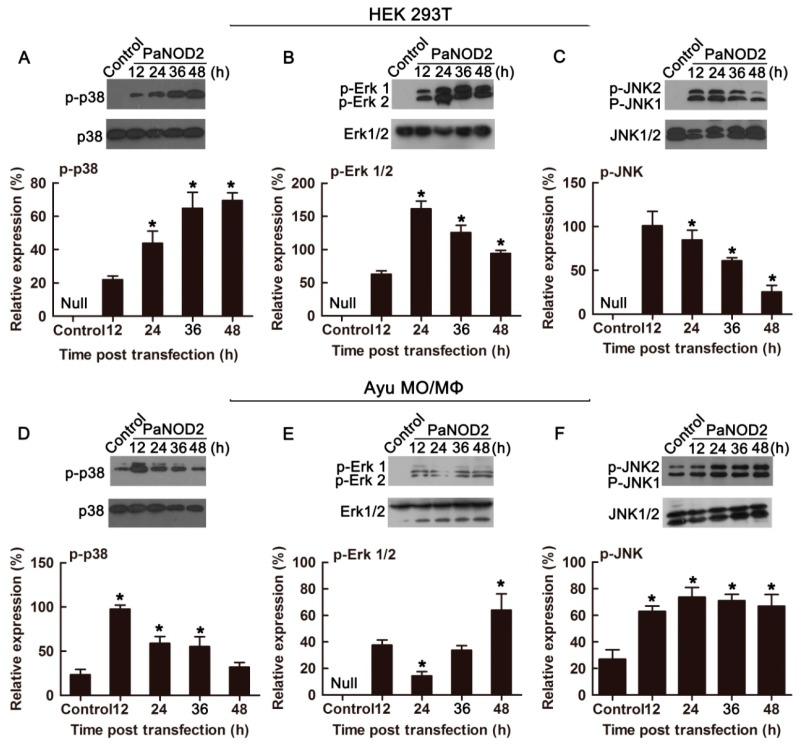
Activation of MAPK signaling by PaNOD2

As another important pathway activated by NOD2 signaling, activation of the MAPK signaling pathway was further examined to verify the conserved role of PaNOD2 in antibacterial processes. The phosphorylation of three conventional MAP kinases (p-38, ERK1/2, and JNK1/2) was examined in both HEK293T cells and ayu MO/MΦ when PaNOD2 was overexpressed, representing the activation of corresponding MAPK signaling. Results showed that the three MAP kinases were not phosphorylated in naïve HEK293T cells, the phosphorylation of which was clearly observed at 12, 24, 36, and 48 h post PaNOD2 transfection (Figure 7A–C). The phosphorylation of p-38 and JNK1/2 but not Erk1/2 was observed in naïve ayu MO/MΦ, but at a relatively low level. After the overexpression of PaNOD2, phosphorylation of p38 was significantly upregulated at 12 hpt, then gradually decreased and returned to the control level at 48 hpt (Figure 7D). Phosphorylation of Erk1/2 was observed at 12 hpt, and reached the highest level at 48 hpt, with a slight decrease at 24 hpt (Figure 7E). Phosphorylation of JNK1/2 was significantly upregulated at 12 hpt and remained at a similar level till 48 hpt (Figure 7F).

HEK293T cells (A–C) and ayu MO/MΦ (D–F) were transfected with pcDNA3.1-PaNOD2 or empty plasmid, with cells collected at 12, 24, 36, and 48 hpt and the phosphorylation of p-38 (A and D), Erk1/2 (B and E), and JNK1/2 (C and F) then examined. Histogram shows changes in the relative band intensity of phosphorylated p-38, Erk1/2, and JNK1/2, with their corresponding total proteins (phosphorylated and non-phosphorylated). Values are means±*SEM*; *: *P*<0.05, **: *P*<0.01.

## DISCUSSION

The NLRs are a large family of cytosolic receptors involved in innate immune responses (Kanneganti et al., 2007; Proell et al., 2008). Bioinformatics have revealed that 23 NLR genes exist in the human genome and at least 34 NLR genes exist in mice (Harton et al., 2002; Kanneganti et al., 2007). The NLR family can be divided into three groups, one of which consists of NOD1 and NOD2, which sense cytosolic peptidoglycan fragments iE-DAP (dipeptide γ-D-glutamyl-*meso*-diaminopimelic acid) and MDP, respectively, driving the activation of the NF-κB and MAPK signaling pathways. The second group consists of NLRs required for the assembly of inflammasomes, leading to the activation of caspase-1 and maturation of IL-1β. The last group consists of the CIITA transcription factor, which is involved in the transcription of genes encoding MHC I (Fitzgerald, 2010; Meissner et al., 2010).

NOD2 is an essential NLR that participates in the recognition of bacterial invasion and viral infection. It initiates the NF-κB and MAPK signaling pathways to induce the production of various proinflammatory cytokines as well as apoptosis and autophagy to eliminate invading pathogens (Shaw et al., 2011). Although the NOD2 gene has been cloned from several fish species, studies regarding its functions and signaling pathways in teleosts are limited, especially the activation of MAP kinases. In the present study, we described the functional characterization of a NOD2 homologue (PaNOD2) in an ayu model. Structural analysis showed that PaNOD2 shared conserved functional domains with its mammalian counterparts, including N-terminal tandem CARD domains, central NBD domain, and multiple C-terminal LRRs. The structural similarity indicates that functional conservation may also exist between PaNOD2 and its higher species homologues.

Previous studies in mammals have shown that, in addition to its role in the activation of cytosolic antibacterial signaling, NOD2 also plays an essential role in the prevention of inflammatory bowel disease (IBD). Three mutations of human NOD2 (R702W, G908R, and L1007insC) are also highly associated with susceptibility to Crohn’s disease in the intestinal tract (Hugot et al., 2001; Strober et al., 2014). In addition, NOD2 is involved in maintaining the equilibrium between the constant exposure of the intestine to various microorganisms and induced host immune responses (Al Nabhani et al., 2017; Balasubramanian & Gao, 2017). Imbalance of this homeostasis can lead to the prevalence of pathogenic bacteria and the explosion of inflammation as well as damage to the intestinal epithelial barrier (Ramanan et al., 2014). It has been reported that a negative feedback loop exists between NOD2 and intestinal bacteria, whereby the accumulation of pathogenic bacteria promotes the expression of NOD2, which, in turn, prevents bacterial overexpansion (Biswas et al., 2012). Furthermore, spontaneous bacterial peritonitis (SBP), a liver cirrhosis, has been attributed to bacterial translocation from the intestine to liver (Wiest & Garcia-Tsao, 2005). Hence, the involvement of NOD2 in maintaining intestinal equilibrium is also likely essential for liver inflammation, albeit indirectly, as susceptibility to SBP is increased in patients with Crohn’s disease (Wiest & Garcia-Tsao, 2005). Furthermore, the upregulation of NOD2 has also been reported during liver injury in mice and humans, and NOD2 may directly contribute to liver injury via a regulatory mechanism affecting immune cells infiltrating the liver and hepatocytes (Appenrodt et al., 2010). As shown in our expression pattern analysis of PaNOD2, the mRNA levels in the liver and intestine were significantly upregulated after *V. anguillarum* infection. Compared to the control group at 24 hpi, the expression of PaNOD2 significantly increased by 111.8- and 241.4-fold in the liver and intestine, respectively, indicating that PaNOD2 may be involved in the maintenance of homeostasis in the liver and intestine. Hence, ayu may also be a potential model for studying IBD and liver injury. However, the detailed mechanisms, signaling pathways, and cells involved still need further elucidation.

Studies in mammals and zebrafish have shown that the LRR motif maintains NOD2 in an autoinhibition state, such that it cannot initiate signaling under physiological state without the stimulation of its ligand MDP (Girardin et al., 2003; Nie et al., 2017). After recognition of MDP by LRR, NOD2 undergoes self-oligomerization and recruits the downstream adaptor receptor-interacting serine/threonine-protein kinase 2 (RIPK2) through CARD-CARD interaction (Park et al., 2007). The activation of RIPK2 subsequently leads to the activation of the IκB kinase (IKK) complex, followed by the translocation of the NF-κB complex into the nucleus and transcription of proinflammatory genes (Rahighi et al., 2009). In contrast, RIPK2 activates transforming growth factor-β-activated kinase 1 (TAK1), which leads to the activation of MAP kinases (Windheim et al., 2007). In the present study, we found that the LRR motif also maintained PaNOD2 in an autoinhibition state, as overexpression of the LRR-deleted mutant, PaNOD2 (ΔLRR), induced more significant NF-κB activation than in the wildtype group in both HEK293T cells and ayu MO/MΦ (Figure 5A, B). In addition, overexpression of PaNOD2 together with its ligand MDP initiated more intense NF-κB activation (Figure 6). Furthermore, overexpression of PaNOD2 led to activation of the MAP kinases, as shown by the phosphorylation of p-38, Erk1/2, and JNK1/2 in HEK293T cells and ayu MO/MΦ (Figure 7). Therefore, the signaling pathways activated by NOD2 are conserved from teleosts to mammals; however, the network of adaptors and series of regulatory mechanisms involved in teleosts still need further clarification.

Collectively, our data suggested the structural and functional conservation of NOD2 receptors in the NF-κB and MAPK signaling pathways between teleosts and mammals, indicating that NOD2-mediated antibacterial innate immunity may have originated early in teleosts and been conserved throughout vertebrate evolution. This study will hopefully provide valuable insights into our understanding of the NOD2-mediated pathways in teleost fish and the evolutionary history of NLRs and associated signaling networks. Notably, NOD2 has been shown to have biological activities aside from as a pattern recognition receptor, including roles in IBD and liver and lung injuries. Therefore, the functional conservation of NOD2 from ayu to mammals may also make ayu a plausible model for studying NOD2-based diseases and therapeutic applications.
